# Our Troubles

**Published:** 1858-05

**Authors:** 


### OUR TROUBLES.

WE have heard a great deal about Editors being hurried for "copy"—pressed almost to death, for something to fill out their paper: thus it is, that often, at the last moment, they have nothing in hand, or in head either. This satisfactorily accounts for the many insufferably stupid, prosy, incorrect editorials in the "leaded" and leaden columns of our hebdominals. *Our* trouble is not of that sort; for, like Mr. BENTON, we are ahead of our publishers, our promises, and ourselves! We had the curiosity, the other day, to count a pile of articles, all corrected, primed, and ready for the printer—such as they are!—and there were over EIGHTY; to say nothing of a dozen or two unfinished ones, broken off in the middle, or at one end, by any one of those irruptions to which all are exposed who have their office or study in their dwellings: irruptions as unprognosticated and unprognosticable as the fiery floods which swept over Herculaneum and Pompeii—playing scatteration to whole hosts of ideas; routing them, horse, foot, and dragoons—good, bad, and indifferent. But, fortunately for us, our ideas (like ourselves) are bendable—bow obediently to the passing storm, then rise, and stand erect as ever—especially as resistance is useless, and is a waste of powder and power.

Another trouble we have: we never can get our articles short enough. We can't talk at all, we exhaust in a minute or two; but, in writing, we can spin out interminably, nonsense and all; and if by any chance we were to be "drawn out" to make a speech, we verily believe we would be neck and neck with the Kentuckian at New Orleans, who bet a Frenchman he could out-talk him: and at it they went; one by one the spectators disappeared; and, by midnight, not a soul was left but the combatants. Next morning, Frenchy was found dead, and the Kentuckian was whispering in his ear.

A good many of our former patients (who are not dead!) take THE JOURNAL; and for any short articles we give them, they may thank themselves, in proportion as they have not the gift of brevity; for we take up one of their old letters, and resolve that we will say all we have to say on the unwritten part. Hence, we literally quit before we are done, causing subscribers frequently to inquire why we don't say more—a compliment, by the way, not paid to many editors in this land; as complimentary as an overheard speech, made of us one dark winter's night, as we were passing homeward in the crowd, which, by some chance, we had just been addressing: "I could sit and hear that man talk all night." Ten years, more or less, before that, a very distinguished politician came to our tent, and said to us, after a speech of certainly not over three minutes long: "You have saved the State!" We were young, then, and felt highly complimented; we felt good all over, reader, for twenty-four hours—when we learned, on reliable authority, that the eminent Senator was drunk. You can't think how we "*hated*" that *truth.* We never before fully understood the meaning of the Scripture expression: "hateth the light," the truth. We love palatable news, and even the bearers of it; and if, in the same sense, a man loves Bible truth—religious truth—we suppose he must be a good man. So much, then, for hating the truth! that the man was drunk—especially as it involved the hating of ourself, for being so egotistically green as not to have seen the state of the case, without the aid of another's eyes. We verily believe "*men are as grass*"—especially the *green* part; and as—peacocks, too.

No regular reader of the *Journal* has, perhaps, failed to notice, that in every number we lug ourselves in. We have two reasons for it. It saves time, and thought, and trouble, and space, to inculcate a truth, by the narration of a fact rather than by the enunciation of a principle; it is clearer, more direct, having the advantage, also, of incapability of being misunderstood. Another reason is, we daily become more conscious of the fact, of how little reliance there is to be placed on any statement which occurs beyond our own personal observation. Not one man in fifty understands any thing an inch beyond his own nose, accurately and critically. About their own immediate business, people manifest considerable practical intelligence, especially as to all the short cuts; but as to things outside of that sphere, the want of intelligence—accurate, practical, satisfactory intelligence—is perfectly amazing, particularly when we take into account the flood of printed matter which is vomited on the American people every day of the world. The truth is, we have too much reading. The rage of the age is to purchase reading matter by the quantity, the bulk; the practical solidity of the subject, being an unimportant consideration: as is well illustrated by the following extract from a letter received, yesterday, from an estimable young lady, a former patient—whom, two years ago, we considered hopeless of recovery, but now writes: "In answer to your printed question, 'How are you?' I reply: I consider myself well; weighing twelve per cent more than for some years. I fear the number of your subscribers here is small. The Gift Enterprises have carried away the common sense of the people. What else could be expected, when the religious part of the community, even, is forward in getting up clubs for purchasing books at such stores. I have been frequently asked to join these clubs, but have uniformly declined, to the surprise of not a few. One says: 'It is not a lottery, and is getting a great deal of reading in the country, which would not otherwise be there.' This 'reading' is of novels, which might be better made a bonfire of. In proof, I called upon a young lady on Monday; 'I read that book through yesterday,' said she, pointing to a gift-enterprise novel. She laughed at my inquiry, if she read such books on the Sabbath! Glad am I that the Journal has taken such a decided stand against fictitious reading."

The more interesting part of this letter, medically speaking, is in proof of how a poor, frail, weak, sickly girl, always complaining, and wholly ignorant of herself, may, in a year or two, by energetic attention to a page or two of written directions, educate her constitution to a habit of health, to which, for years, she had been a stranger—without medicine almost, but with a little proper instruction.

"I have had but one slight cold all winter, that affected my throat; but, with a little attention, it passed off in a few days. I weigh more than I have done in several years. Appetite good, sleep soundly. Feet warm, and regular in all my habits. Have not had *sick headache* in a year. Before I consulted you, if I accidentally scratched or pricked myself, it would fester and be very sore; now, it will be a little red, and in a day or two, will be well. Then, my blood was dark, now, it is crimson. I have sometimes been laughed at, for keeping my mouth shut, when going out in the cold, as you advised me. My friends say to me: '*What is the use of being so careful, you are well*

*now?*' I tell them, that is the way to keep well. I hope I have grown a little wiser within a year or two. I used to think I was careful of my health; but it was inattention to little things which first affected it. I now bid fair to live as old as grandfather, who is eighty-eight, is well and hearty, takes care of his own garden, saws his own wood, always has been an early riser, and usually has early vegetables before his neighbors: his garden is his delight; he says that exercise is just what he needs to keep him well in his old age. Grandmother, also, enjoys good health, in her eighty-fourth year, and they have lived together sixty-nine years; she was but fifteen, and he not quite twenty, when they were married. I think that is carrying out *your* idea of 'marrying young.' I once told grandmother, I thought she married too young. 'Why,' said she, 'people married much younger then than now; and had I to do it over again, I would have gone backward in that respect."

"Why, Doctor, when you advised 'single folks' to marry, why didn't you put it *single men*, for what have the women to do in the matter? Would you have them go out into the world and look for husbands? My cousin tells me I am less indoor than out; and if I were to marry, I might have to give up my horse."

There, my lady readers, there is a lesson for you worth remembrance. There is a lady whom we persuaded to horseback exercise two years ago. She has learned to love it; and chooses to forego a husband rather than lose her horse. Horseback exercise—the best substitute for a pill a young lady ever took; for, besides its healthful influences, it is the best educator to activity of body, to sprightliness of mind, to courage, and to self-reliance, that we know of. Why don't the residents of Fifth Avenue, Fourteenth street, and Union Square, spend more of their money at DISBROW'S, and less at the spa, the theatre, and the vulgar fancy ball, and its twin sister, the "Charity Fete." Let our daughters be amused on horseback, not by a maudlin novel; let them learn to sit on a hard saddle, instead of lolling on the velveted lounge; let them snuff up the fresh air of the morning, instead of poisoning their blood in the stifling atmosphere of furnace-heated parlors. Away with this insipid, helpless inactivity, as ruinous to body as it is to mind. When our daughters read books, let them be such as will compel them to think, to think closely, logically; for the grand object of all primary studies is to teach the young how to think with vigor—to think to purpose, there must be bodily health, the substratum, the essential foundation of force of character. And more, to be straight as an arrow is an accomplishment which nothing so well secures as the saddle; and no slight connection is there between erectness of manner and of morals—just as there is no slight connection between purity of character and purity of person.

To horse, to horse, then, young ladies, and away. The character, be it man or maid, which glories in joyous and abundant horseback riding, cannot be insipid, must have force, vigor, energy; must be lovable in a woman, and command respect in men, and very likely have, at least, some of that ennobling quality, truthful candor, which our fair correspondent exhibits at the close of her letter, the last half of which is kept back, when she says, "I agree with you, Doctor, that 'it is a good thing to have everything to do, and more too.' I am a great aunt; I am constantly employed in the old homestead, where five generations on mother's side have lived; and it is good for my health. I do a little of almost everything: take charge of the house, and transact business for father, and see that all things are kept straight. Yet, at times, there seems to be a longing for companionship, a desire for a worthy object on which to place the affections; yet I cannot be wedded to splendid misery—but to one only who has a pious heart, and is possessed of high moral principle." There, reader, is true nobility. That young girl is by birth a princess. Where is the man who is worthy of her?

On reflection, we have missed being short this time. But our head is so big and full, we have apprehensions sometimes that, if opened, there would be found in it nothing but wind and water—a spring of puffy insipidity, except about "Consumption," and things of that sort; and as to that, both in speech and act, we are in the category of the countryman, who couldn't cure *a-goose* (agues), but was death on fits.

---

OLD DOMINION COFFEE POT, we personally know to be a philosophy, a luxury, and an economy—from $1.50 to $5.—ARTHUR BURNHAM & GILROY, Philadelphia.

"A WOMAN'S THOUGHTS ABOUT WOMAN." *Budd & Carlton*, 310 Broadway, New York. $1. These thoughts by MISS MULOCK, an English Lady, are at once severe, kind, just, and practical, and merit every woman's reading and digestion.

